# Property Graph vs RDF Triple Store: A Comparison on Glycan Substructure Search

**DOI:** 10.1371/journal.pone.0144578

**Published:** 2015-12-14

**Authors:** Davide Alocci, Julien Mariethoz, Oliver Horlacher, Jerven T. Bolleman, Matthew P. Campbell, Frederique Lisacek

**Affiliations:** 1 Proteome Informatics Group, SIB Swiss Institute of Bioinformatics, Geneva, 1211, Switzerland; 2 Computer Science Department, University of Geneva, Geneva, 1227, Switzerland; 3 Swiss-Prot Group, SIB Swiss Institute of Bioinformatics, Geneva, 1211, Switzerland; 4 Department of Chemistry and Biomolecular Sciences, Macquarie University, Sydney, Australia; University of Rome Tor Vergata, ITALY

## Abstract

Resource description framework (RDF) and Property Graph databases are emerging technologies that are used for storing graph-structured data. We compare these technologies through a molecular biology use case: glycan substructure search. Glycans are branched tree-like molecules composed of building blocks linked together by chemical bonds. The molecular structure of a glycan can be encoded into a direct acyclic graph where each node represents a building block and each edge serves as a chemical linkage between two building blocks. In this context, Graph databases are possible software solutions for storing glycan structures and Graph query languages, such as SPARQL and Cypher, can be used to perform a substructure search. Glycan substructure searching is an important feature for querying structure and experimental glycan databases and retrieving biologically meaningful data. This applies for example to identifying a region of the glycan recognised by a glycan binding protein (GBP). In this study, 19,404 glycan structures were selected from GlycomeDB (www.glycome-db.org) and modelled for being stored into a RDF triple store and a Property Graph. We then performed two different sets of searches and compared the query response times and the results from both technologies to assess performance and accuracy. The two implementations produced the same results, but interestingly we noted a difference in the query response times. Qualitative measures such as portability were also used to define further criteria for choosing the technology adapted to solving glycan substructure search and other comparable issues.

## Introduction

Nowadays the use of high throughput technologies and optimized pipelines allows life scientists to generate terabytes of data in a reduced amount of time and subsequently feed quickly and comprehensively online bioinformatics databases. In this scenario, the interoperability between data resources has become a fundamental challenge. Issues are gradually being solved in applications involving genome (DNA) or transcriptome (RNA) analyses but problems remain for less documented molecules such as lipids, glycans (also referred to as “carbohydrate”, “oligosaccharide” or “polysaccharide” to designate this type of molecule) or metabolites.

Glycosylation is the addition of glycan molecules to proteins and/or lipids. It is an important post-translational modification that enhances the functional diversity of proteins and influences their biological activities and circulatory half-life. A glycan is a branched tree-like molecule that naturally lends itself to graph encoding. However, glycans have long been described in the IUPAC linear format [1], that is, as regular expressions delineating branching structures with different bracket types. Such encoding can generate directional/linkage/topology ambiguity and is not sufficient in the handling of incomplete or repeated units. More recently, several encoding formats for glycans have developed based on sets of nodes and edges, e.g., GlycoCT [2], Glyde-II [3,4], IUPAC condensed [5], KCAM/KCF [6,7] or more recently WURCS [8]. To date the GlycoCT format is acknowledged as the default format for data sharing between databases [9] and consequently the most commonly used format for storing structural data. Glycans are composed of monosaccharides (8 common building blocks and dozens of less frequent ones as described in MonosaccharideDB (http://www.monosaccharidedb.org) that are cyclic molecules. These monosaccharides are linked together in different ways depending on carbon attachment positions in the cycle as detailed further.

**Fig 1:**
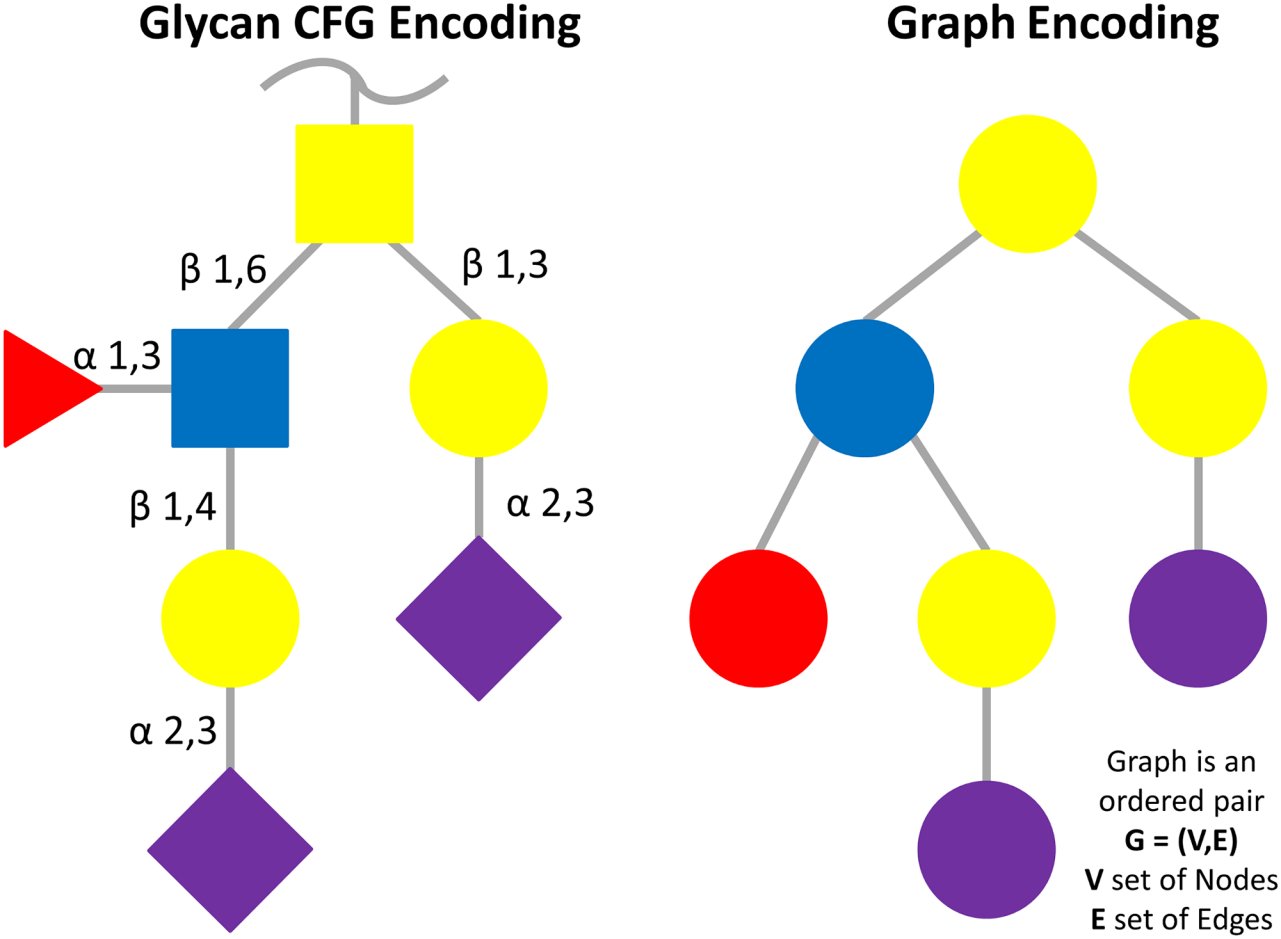
Glycan CFG encoding and graph encoding. On the left hand side a glycan structure encoded with CFG nomenclature is presented, while the right hand side shows the same structure translated into a graph. Each monosaccharide or substituent becomes a node and each glycosidic bond becomes an edge in the graph. Avoiding any loss of information all the properties of each monosaccharide or substituent are converted in node properties whereas glycosidic bond properties are translated in edge properties. To be more clear the colour code associate with the monosaccharide type is preserved among the images.

In a graph representation of a glycan, each monosaccharide residue is a node possibly associated with a list of properties and each linkage is an edge also potentially associated with a list of properties. In fact, chemical bonds between building blocks, called glycosidic linkages, are transformed into edges in the acyclic graph structure. An example is shown in Fig 1, where the simplified graphic representation popularised by the Consortium for Functional Glycomics (CFG) [10] (originally proposed by the authors of Essentials in Glycobiology [11]) is matched to a graph. This notation assigns each monosaccharide to a coloured shape (e.g., yellow circle for galactose, shortened as Gal). Shared colours or shapes express structural similarity among monosaccharides. For example, N-Acetylgalactosamine (yellow square) differs from galactose (yellow circle) through a so-called substituent (removal of an OH group and addition of an amino-acetyl group). “Substituent” as a property is precisely the type that qualifies a node.

Starting with the CarbBank project in 1987 [12], a range of glycoinformatics resources containing glycan-related information has been developed thereby creating a variety of reference databases [13–15]. In the last two years, two articles [16,17] have been published proposing mechanisms for connecting glycan-related (or glycomics) databases. The respective authors suggested moving towards new technologies designed for semantic web, which are adapted to aggregating information from different sources. The outcome for these proposals was a common standard ontology called GlycoRDF [16] that is now being widely adopted by the community to develop glycomics resources that cooperate and share standard formats enabling federated queries [9]. Nonetheless, in an effort to confirm the relevance of RDF as the technology of choice for glycomics data representation and integration, comparison with other graph-based technologies such as Property Graph is necessary. In this paper we discuss methods for interconnecting different databases through addressing the question of glycan substructure (motif or pattern) searching. This report should be considered as a preliminary study of feasibility and a comparison between different software implementations. Our main goal is to compare the performance of these two technologies (RDF and Property Graph) for glycan substructure search. In this study, Neo4j was chosen as a representative option for Property Graphs and multiple RDF triple stores were tested mainly due to the shared graph query language. Specifically, we have used for the latter: Virtuoso Open-Source Edition [18], Sesame [19], Jena Fuseki [20] and Blazegraph [21].

Searching (sub)structures is meaningful in glycomics. It matches the concept of glycan epitope or glycan determinant associated with the part of the whole structure that is recognized by glycan-binding proteins, which include lectins, receptors, toxins, adhesins, antibodies and enzymes [22]. Several substructure search solutions have been developed including for instance, regular expression matching [23] as applied to processing glycans in linear format (IUPAC) [5]. Although these approaches have showed reasonable robustness, they also have intrinsic limitations especially in handling structural ambiguities. Frequently, experimental data is insufficient to assign a precise monosaccharide (e.g., Galactose) to a position in the structure so that it is characterised only by its carbon content (e.g., Hexose). Regular expressions do not account for dependencies while a graph description handles inheritance of properties (e.g., Hexose -> Galactose). Emerging technologies capable of storing graph models can be used, namely, Resource Description Framework (RDF) [24] and Property Graph databases [25]. Even though RDF and Property Graph databases can be used to reach the same goal, these two approaches are not synonymous and can be distinguished. RDF is designed for storing statements in the form of subject–predicate–object called triples, whereas a Property Graph is designed to implement different types of graphs such as hypergraphs, undirected graphs, weighted graphs, etc. Nonetheless, all possible types of graphs can be built with triples and stored in an RDF triple store [26]. Furthermore, Property Graphs are node-centric whereas RDF triple stores are edge-centric. For this reason, RDF triple stores use a list of edges, many of which are properties of a node and not critical to the graph structure itself. Moreover, Property Graph databases tend to be optimized for graph traversals where only one big graph is present. With RDF triple stores, the cost of traversing an edge tends to be logarithmic [27]. Finally, the query language is another key point in the comparison: RDF triple stores support SPARQL [28] as a native query language whereas Property Graphs have mainly proprietary languages. Even though Neo4J has a plugin for SPARQL, it relies essentially on its own proprietary language called Cypher [29]. In either case, a substructure search can be defined with the query language provided by these new technologies. To reach our benchmarking goal in this application, we first describe the main steps of building substructure search software using alternatively an RDF triple store or a Property Graph databases. We then provide a selection of quantitative and qualitative measures investigated during the study such as portability. Finally, we summarise the main achievements and draw some conclusions on the expected characteristics of a web application for glycan substructure search.

## Material and Methods

The development of a software solution to perform glycan substructure searching involves two main tasks 1) the storage of glycan structures and 2) translation of a query into a specific query language for pulling out all the glycan structures that match the query pattern.

The glycan encoding and the data storage sections provide details regarding these tasks using native graph stores. The substructure search query shows how specific languages provided by RDF and Property Graph databases can be used.

### Data structure

As previously described most dedicated glycan databases store structures using string encoding standards. This multiplicity led to create a new layer between the data store and the glycan encoding formats to decouple the glycan structure information from any string-encoding format.

A comprehensive framework was developed within the EUROCarbDB project for parsing multiple glycan encoding formats [30]. Even though this framework is still used and includes parsers for each glycan encoding format, for pragmatic reasons we have relied on an in-house library, called MzJava [31]. It is an open source library that provides a data structure and specific readers for multiple glycan encoding formats that are not limited to processing mass spectrometry data. The MzJava data structure organizes the information from a glycan structure into a directed acyclic graph, which can be directly stored into a graph storage solution. Monosaccharides, the basic units, which compose the glycan structures, are treated as graph nodes and all the biological properties of these building blocks are stored as separate node properties. Substituents—particular building blocks, which can be combined with basic units—are handled separately from monosaccharides and are represented as extra nodes in the data structure.

All glycosidic linkages are transformed into edges in the acyclic graph structure. Other edges are introduced for every linkage between monosaccharides and substituents, so-called substituent linkages. Each edge carries a list of properties, which identify different chemical aspects of the bond itself. Each glycan is represented by a set of nodes selected between available monosaccharides and substituents and a set of edges, which connect the nodes (Fig 1). The direct acyclic graph used by the Mzjava data structure can be directly loaded into a native graph store.

### Data Storage

The data store interacts with the data structure layer that was introduced in the previous section. Native storage of the direct acyclic graphs used by the data structure layer was the first main requirement for the data store. Because both RDF and Property Graph technologies fulfil this requirement, we have developed two different data stores using each of these technologies.

For populating the data stores, a collection of known glycan structures was extracted from GlycomeDB [13], the largest repository of known glycan structures publicly available. It contains more than 34,000 structures collected from different online resources. The GlycoCT version of GlycomeDB [13] was used in this study to compare the performance of RDF versus Property Graph and the MzJava reader used to translate all structures into the supported data structure, which were then stored into both RDF triple store and Property Graph data stores (see “Glycan encoding” section). All the structures with repeats, underdetermined regions or containing not fully characterised monosaccharides were omitted from this study in order to produce consistent results with each query and ease comparison between the two implementations. In the end, the data store contained 19404 possibly redundant glycan structures (redundancy comes for instance for the same structure identified in two different species since at this stage, we do not account for taxonomy). Each structure was treated as a single graph, meaning that both implementations contain 19,404 disconnected graphs. In the end, the dataset contained 233,633 distinct nodes divided in 19,404 different graphs, with an average of 12 nodes for each graph. Here, the largest is composed of 63 nodes other details about the distribution of nodes among the structures are provided in S10 Table.

Details about the development of the two data storage implementations are illustrated in the following sections.

### RDF implementation

**Fig 2:**
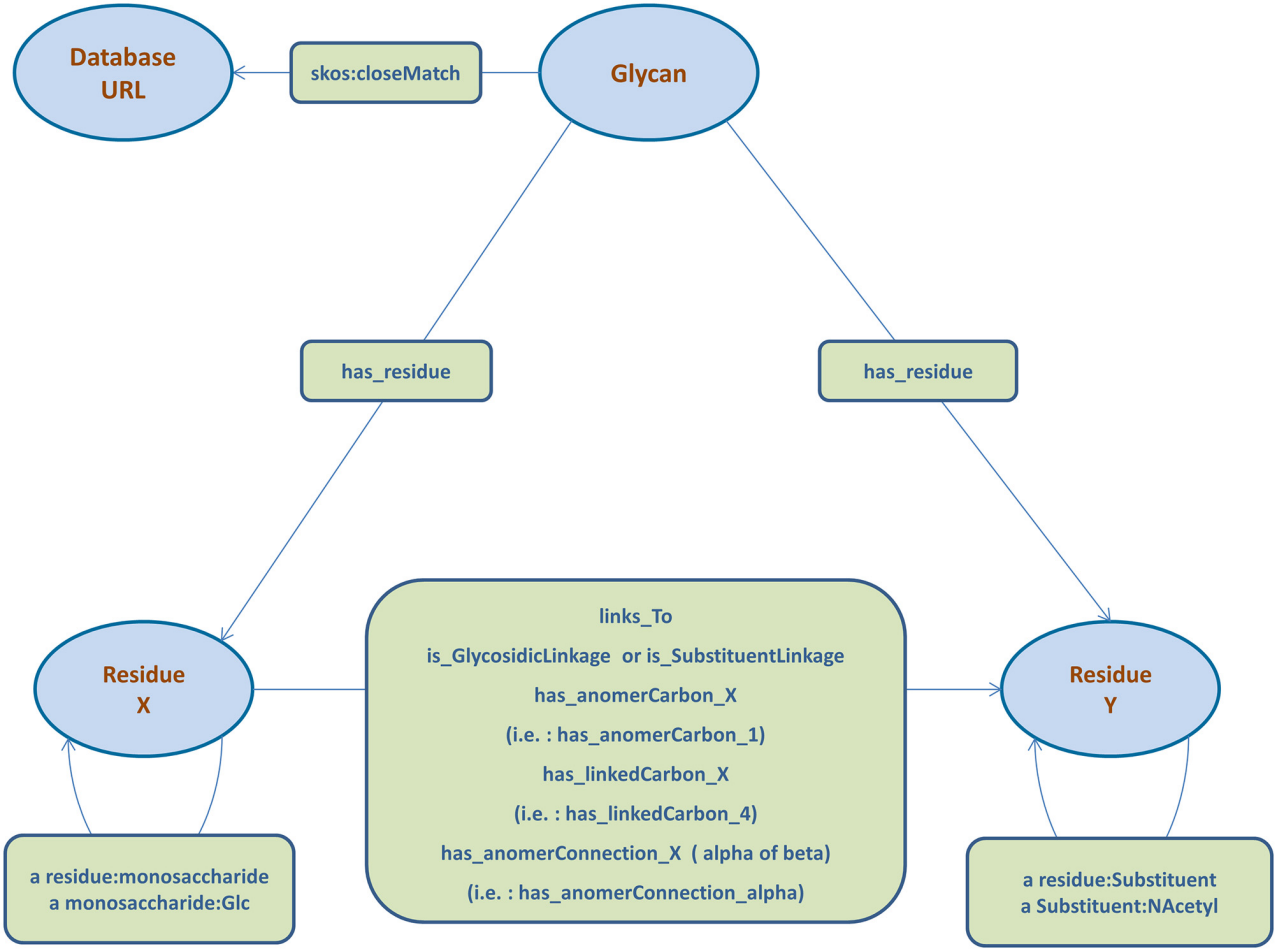
Ontology overview. Overview of the ontology developed for translating glycan structures into RDF/semantic triples. The figure shows all the predicates and the entities used for defining a glycan structures into the RDF triple store.

RDF can only deal with triples, statements in the form subject-predicate-object. For this reason, it is necessary to develop a model that translates a glycan structure with all potential biological properties into a list of triples. Fig 2 shows how this model can be used for a simple structure with a monosaccharide and a substituent.

The proposed model is based on the GlycoCT standard where the structure is encoded in a residue list and a connectivity list. All monosaccharides and substituents are treated as separate components and annotated in the residue list with a specific ID. The connectivity list contains the linkages between the components annotated in the residue list. Our model follows the same principle: all substituents are treated as separate components as opposed to merging them with their associated monosaccharides in order to avoid contaminating the model with biological assumptions.

“Glycan” is the first entity in the model and it has two predicates associated: “closematch” taken from the SKOS ontology and “has_residue”. The “closematch” predicate connects the “Glycan” entity with URLs that identify the specific glycan in other databases. In this way, references to external databases can be kept in our model for refining the search. The user can always add alternative references using the same predicate. The “Glycan” entity links each monosaccharide and substituent to the “has_residue” predicate, grouping together all the building blocks that belong to the same glycan structure.

Every “Residue” entity represents a particular monosaccharide or substituent, so the number of components for each structure is equal to the sum of monosaccharides and substituents. Chemical properties related to the components can be stored using RDF:property. In this model we provide “residue:monosaccharide” and “residue:substituent” as RDF:type for specifying the type of components and properties like “monosaccharide:Gal” or “substituent:NAcetyl” can be used for defining the actual molecule.

All predicates available are shown in Fig 2 whereas the list of all monosaccharides and substituents already defined in the ontology can be found in S11 Table. The user can extend the ontology adding new predicates or property to encode supplementary information regarding nodes.

Component entities are connected to each other following the actual pattern of the glycan structure. In other words, a triple with a “links_To” predicate is added to the triple store for each linkage between two different components. In this ontology no entity represents linkages, we rely on multiple triples for storing the specifications of a linkage. Each time a piece of information about a specific edge is added, a new triple with the parent node as subject and the child node as object is inserted in the triple store. The information is encapsulated in the predicate itself. The ontology provides the user with several predicates for describing each piece of information related to the linkage.

Starting from the root node every linkage between a parent component and a child is added to the triple store following a breadth first search algorithm. Thus we prevent any possible edge duplication in the graph.

In the end, each glycan structure stored in the RDF triple store is composed of at least a “Glycan” entity associated with many different “Residue” entities.

By introducing a predicate for each linkage and component property, we allow the stratification of information. The potential loss of performance due to spreading information on different layers is justified by our wish to preserve approximate search options (e.g. search with missing information or tolerating mismatches). Indeed, merging all properties within a predicate lacks the flexibility that is crucial for glycan substructure search tolerating fuzzy matches.

### Neo4j implementation

The Java API provided by Neo4j was used for storing glycan structures into a Property Graph. Each structure has been added to the same graph and the glycan structure ID has been stored in all the nodes related to a particular structure. The final result is a disconnected graph where structures can be grouped by ID. Spreading the glycan ID, as opposed to keeping it in the root node, is useful to quickly retrieve the identifier when the substructure does not include the root node. In finer details, each monosaccharide or substituent has been added to the graph database as a node whereas each linkage is encoded as a relationship between two nodes. To encode biological properties of components or linkages we have used respectively node and relationship properties. Property Graphs can directly store graphs including nodes and edges properties.

### Substructure Search Query

Starting from a substructure query, each data store implementation is queried in order to retrieve all glycan structures that contain the query pattern. In fact, there is no difference between a complete structure and a substructure in terms of encoding format. Consequently, the workflow presented in the Glycan encoding section can be used for extrapolating and organizing the substructure information into a common data structure. Then the direct acyclic graph is translated into a technology specific language for performing a graph pattern search among the structures contained in the data store. The languages provided by RDF and Property Graph databases describe a graph pattern and find all the graphs which contain it, and support our software solution for the retrieval of all the structures in the data store that contain a query substructure.

The following illustrates the process of translating the data structure graph into specific RDF and Property Graph databases query languages.

### RDF SPARQL Query

**Fig 3:**
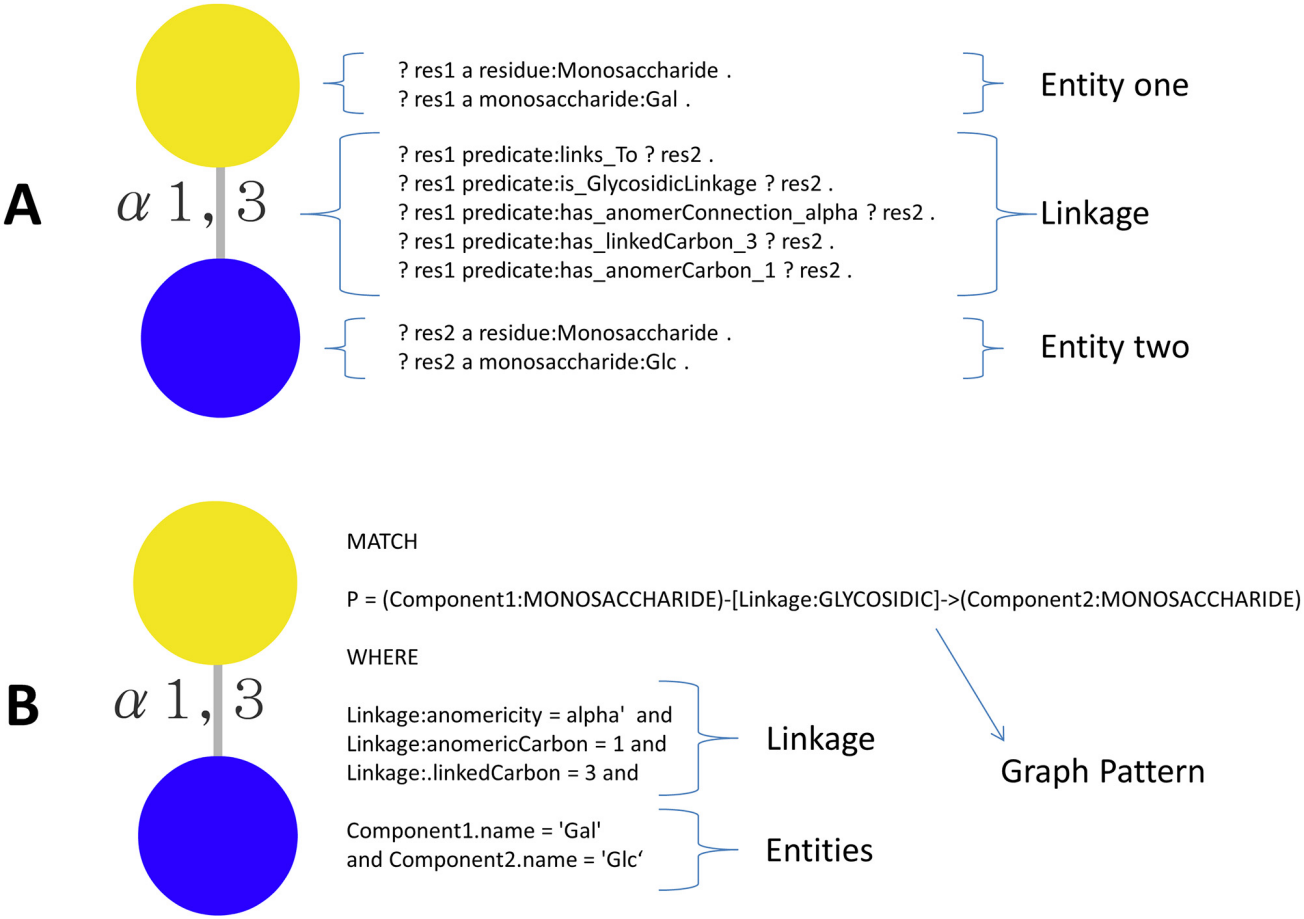
Query building example. A. Example of use of the RDF model to build a SPARQL query from a glycan substructure focussing on the translation process. The prefix part of the query is omitted but further detailed examples are provided in the S1 File. B. The same example is shown with building a Cypher query, the native language in Neo4J. Similarly, additional examples are provided in the S1 File.

Following the ontology described in Fig 2, glycan substructures can be translated into a SPARQL query. A native support to this query language is provided by each RDF triple store, thereby not tying our solution to any particular product, however, the queries support SPARQL 1.1 [32]. Fig 3A shows the translation process on a substructure where a galactose residue is connected to a glucose with an alpha 1,3 linkage. Every monosaccharide or substituent becomes an entity and each property is encoded in one of the predicates or the properties described in the model. Sesame API [19] together with the appropriate JDBC driver has been used for querying Virtuoso Openlink, Blazegraph, Sesame and Jena Fuseki. Detailed examples of larger structures are provided in the S1 File.

### Neo4j Cypher Query

In order to interact with the database, Neo4j provides a native query language called Cypher. The translation process of a glycan substructure into a Cypher query is shown in Fig 3B. Two main parts are present in the query: the graph pattern and the property specification. The first part delineates the shape of the substructure while the second specifies the properties of each node or edge. In addition to Cypher, Neo4j provides a native object access Java API to interact with the database. Cypher is known to be slower than the native object access [33], however version 2.2 has improved performance and comes with a new cost-based query optimizer.

### Setup

All tests were performed using a Dell Precision T7400 with the following hardware features:

- CPU: Intel(R) Xeon(R) X5482
- RAM: 56 GB DDR2 ECC
- Hard disk capacity: 500 GB
- Operating system: Linux Cent OS version 6.6 64-bit
- Java JDK version: 8
- RDF triple store: Virtuoso Open-Source Edition version 7.2, Sesame 4.0, Jena Fuseki v2, Blazegraph 1.5.3
- Property Graph: Neo4j Community version 2.2

For Virtuoso, NumberOfBuffers and MaxDirtyBuffers were changed to 170,000 and 130,000 respectively. Moreover, the MaxCheckpointRemap was adjusted according to the size of our database. A further increase of the buffers reported the same performance. Neo4j embedded version was preferred over the REST implementation. The embedded version has the advantage of removing any latency introduced by the REST communication between the service and the server. Moreover, it can be accessed directly from the java application with a specific API provided by Neo4j.

For building the Neo4j instance, Node_auto_indexing and Relationship_auto_indexing have been activated. In addition, we have set the cache_type option to “strong” in order to keep the whole database in RAM.

We have used Java JDK version 8 to run Neo4j, Blazegraph, Jena Fuseki and Sesame setting the heap size to 8 GB.

## Results and Discussion

In an effort to compare the usage of Property Graph vs. RDF triple store to address the substructure search problem, we built a dataset with 19,404 glycan structures extracted from the glycan structure repository GlycomeDB and compared the average query time of two data sets described in S8 and S9 Tables. The size of the queries have been divided into five groups:

- Very short: less than 5 residues
- Short: between 5 and 15 residues
- Middle: between 15 and 25 residues
- Large: between 25 and 35 residues
- Very large: more than 35 residues

The first set contains 128 queries (S8 Table) that represent biologically relevant use-cases and have been identified as glycoepitopes, that is, parts of glycans recognised by glycan-binding proteins. This list was obtained from the GlycoEpitope database [34] and further substantiated by information reviewed in [22]. Glycoepitopes are limited in size, i.e. between 2 and 13 residues but approximately 70% of these substructures contain between four and seven residues. This reference set is important for the future implementation of a web application that will perform substructure search for glycobiologist users. It contains mainly short and very short queries.

The second set (S9 Table) contains 60 queries. The structures were randomly picked in GlycomeDB, with the prerequisite of spanning between 25 and 60 residues. This set was exclusively created for benchmark purposes and contains only large and very large queries. There is no biologically relevant relation in these substructures, but this data set pushes the software to its limits and tests the reliability of different implementations of RDF triple stores and Property Graphs.

### Quantitative Measures

At first an empty query was performed to initiate the test environment and then each query structure, present in S8 (first set) and S9 (second set) was run 10 times and the average query time was calculated for the last nine queries. All time is measured in seconds (s).

The results (S1 Table) for the first set of queries show that the Virtuoso RDF triple store and Blazegraph have a better query response in 95% of the queries, the other 5% is cover by Sesame. Neo4j, as the only representative of Property Graph, only performed faster than Virtuoso for 12 queries (Ids 49 to 52 in S1 Table) and never exceeded the performance of Blazegraph. The average query time through the whole set of queries shows that Neo4j performance is comparable with Sesame but is still 0.8 s slower than Blazegraph.

S2 Table shows the results for the second query set that were technically more instructive. Virtuoso Triple store column is empty because we could not run the benchmark with this RDF triple store. Using the machine described in the setup, we have tested both the development and stable versions of Virtuoso and none of them produced an output result. However, we observed two different behaviours: using the stable version, the machine froze after submitting the query until Virtuoso crashed. With the development version, we were not able to get any query results after hours of computation. We attempted to solve this problem while setting to 5 the swappiness of the operating system, to no avail. In the end, we contacted the Openlink support but have not yet received an answer. RDF technology is relatively new and some implementations still need debugging or meet scaling issues.

**Fig 4:**
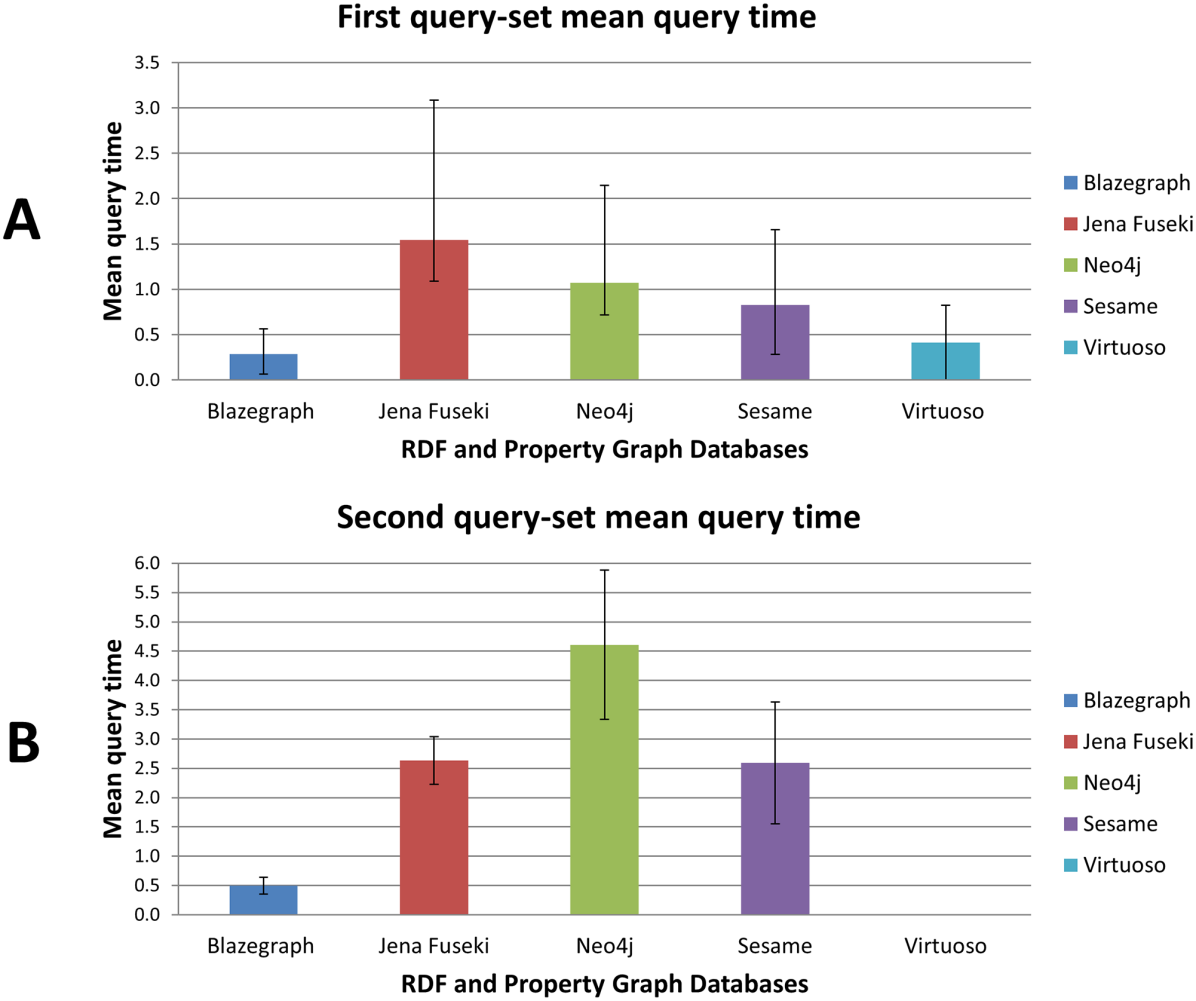
Average query time. The mean value calculated on the response times of each query in both sets is shown in two bar charts. Panel (A) shows the mean query times for the first set and panel (B) contains the values for the second set. The column assign to Virtuoso in the second set of query is empty because we could not record any data due to a problem in running large and very large queries.

**Fig 5:**
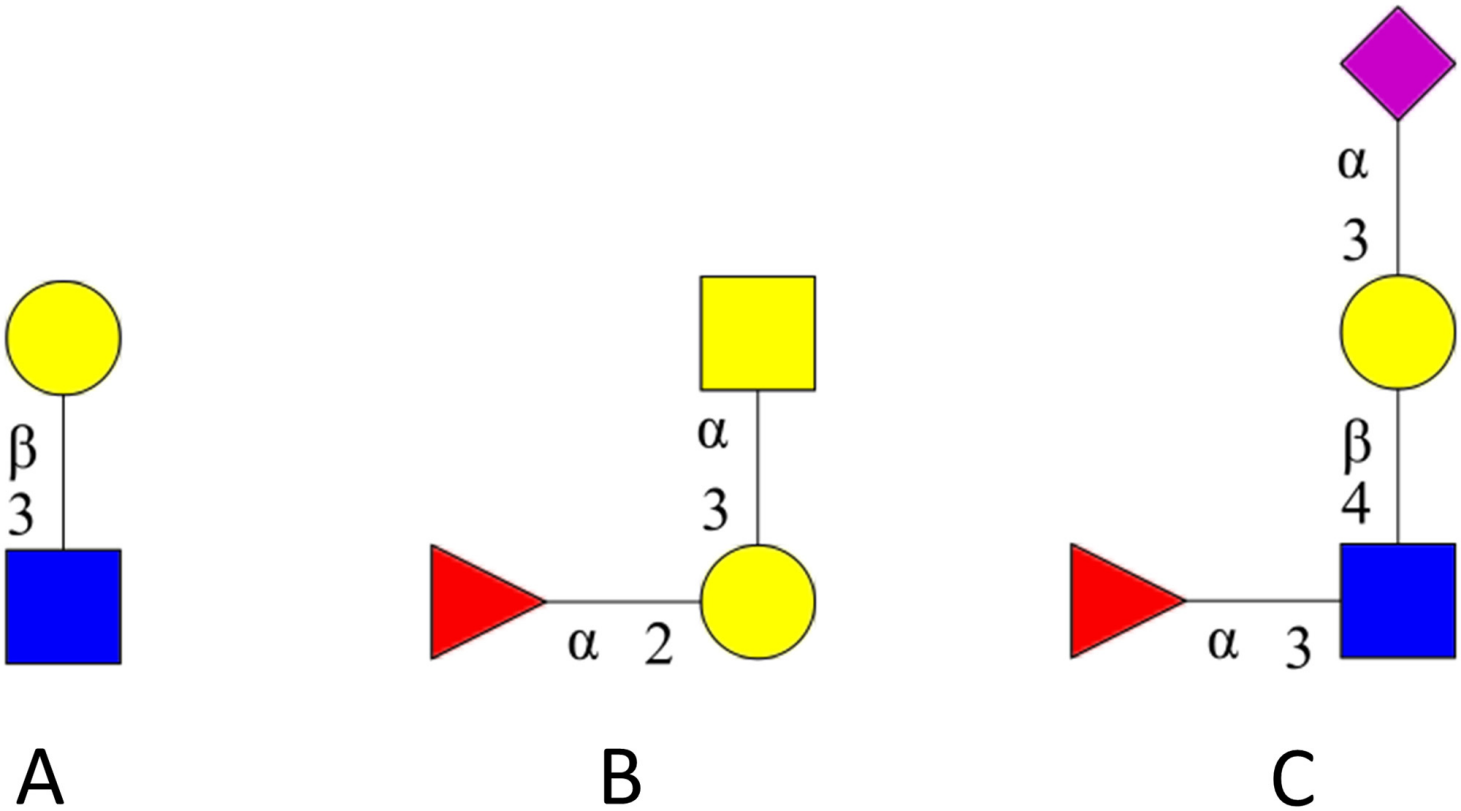
2D structure of query glycoepitopes. The 2-dimensional structure of three well-known glycoepitopes listed in Table 1, namely (A) Lactosamine Type One. (B) Blood Group A. (C) Sialyl Lewis X is shown. Response time for each is shown in Table 1.

When compared to the RDF triple stores, Neo4j slowed down as the size of the queries increased. In this case, the difference between Neo4j and Blazegraph, calculated on the average query time in the whole set, is close to 4 seconds. Surprisingly Jena Fuseki, the slowest database in the first benchmark, is almost 2 second faster than Neo4j. The average response time was calculated for each query in both datasets and the results are summarised in Fig 4. The average query response time of three glycan determinants shown in Fig 5 is detailed in Table 1. Complete information regarding the results obtained with the two sets is provided in the S4 and S5 Tables.

**Table 1:** Comparison of average response query time for 3 glycoepitopes (see Fig 5). A comparison of the average query time of Property Graph and RDF triple store databases tested in this study (columns) for three well known epitopes (rows). SPARQL and Cypher queries for these glycoepitopes are provided in the S1 File.

	Virtuoso	Neo4j	Sesame	Jena Fuseki	Blazegraph
**Lactosamine Type One**	0.114 s	0.827 s	0.814s	0.941 s	0.495 s
**Blood Group A**	0.104 s	0.945 s	0.817 s	0.869 s	0.225 s
**Sialyl Lewis X**	0.469 s	1.012 s	0.706 s	1.970 s	0.120 s

Contrary to our initial intention, we have not fully tested Neo4j with SPARQL since the supporting third-party plugin runs approximately 2.4 to 27 times slower than Jena Fuseki [35], which is already the slowest RDF triple store in our benchmark. Dealing with a large disconnected graph is the key point of the discussion. Property Graphs are in general optimised for storing large connected graphs, for example friend-of-a-friend networks. The data structure is designed to efficiently perform a graph traversal that computes the shortest path between two nodes or retrieves all the friends of a friend. These conditions are not satisfied in our substructure search problem where the graph is a collection of small and disconnected graphs. For each query, the Property Graph database performs a full scan of the collection searching for a specific pattern. In other words the software engine has to search the root of each structure and start the traversal. This matches the use case guiding Neo4j’s implementation and design but not the specificity of glycan substructure search.

In contrast, RDF is an information representation with different technological implementations. In most RDF triple stores, a single table of four columns holds one quad, i.e. triple plus graph identifier, per row. When a specific pattern is searched in the structure collection, the engine retrieves all the glycan entities and runs a traversal on each one. The process is sped up by the use of entity indices that allows the RDF engine to find the next structure more efficiently than a Property Graph database.

In the end, graph pattern searching as implemented by RDF databases appears to match more closely the glycan substructure use case, which (in the absence of bugs) explains why this type of graph database performs well on our benchmark.

All the results divided by specific methods can be found in supporting materials S3–S7 Tables.

### Qualitative Measures

We provide qualitative measures concerning the query language, the ease of usage and the level of support. Although difficult to evaluate, these criteria are significant for choosing which type of technology to use. In the last part we underline some cross-platform issues related to the Virtuoso triple store.

#### Query language

As described above RDF triple stores support the World Wide Web Consortium (W3C) endorsed SPARQL standard as the common query language. Consequently, the proposed (sub)structure search can be used by different RDF implementations without changing the application. In comparison, Property Graphs are language specific relying on proprietary languages such as Cypher (Neo4j), which potentially limits application portability and requires drastic change in the software when moving to alternative implementations.

The W3C promotes the development of high-quality standards, making SPARQL a good choice for production software. In the case of Neo4j, there are add-on components that allow this Property Graph to be accessed as a triple store and potentially queried with SPARQL. As mentioned earlier, third-party contributors implemented these plugins and there is no guarantee of future development and support. In this study, we have not tested these plugins mainly because they have poor performance [35]. SPARQL is translated into a native object access API introducing latency in the query process and negatively impacting performance.

Finally, providing a standardised query language gives RDF an important advantage, especially when the stability of the software technology used is crucial.

#### Level of support

Although RDF and Property Graph databases were recently introduced, they have lively communities behind them showing the need for natively storing graph data. RDF and SPARQL standards have extensive support both from the W3C and the triple store vendor websites. Google groups and user groups are available for supporting the development and discussing issues for both technologies.

The Property Graph community compared to that of RDF is fragmented due to the lack of standards in query language and API. Most of the support for Neo4j and Cypher comes from the online manual available on the company website. With each new release, the manual is updated and explains how to get the maximum performance out of this database. Despite a detailed documentation and multiple tutorials provided by the Neo4j site, the query language lacks support from other industry partners. Three different query languages were proposed for Neo4j in the past 11 years and the current Cypher may not be the last.

The RDF community instead is led by the W3C that periodically updates the standard following community suggestions. All implementations of RDF triple store have to follow the W3C recommendations. The actual availability of multiple triple store implementations gives the RDF community more stability compared to Neo4J. Moreover, Open Data [36] and Linked Data movements are pushing the usage on RDF and SPARQL as a way of interconnecting online resources. In bioinformatics, some online resources such as UniProt [37] are already accessible through SPARQL. Regarding the four RDF triple stores tested in this study, each vendor website provides useful information about setting up the environment and tweak the settings.

In the end, the possibility of connecting multiple online resources and the stability shown by the RDF community place RDF in a favourable position compared to Property Graph.

#### Ease of usage

The embedded version of Neo4j databases can be easily added to a java project through a jar or a dependency. The API for building the graph is simple and intuitive. A great advantage is provided by the option of directly adding properties to nodes and edges.

Theoretically speaking Neo4j and Property Graph databases fit our problem better than RDF, nevertheless they are not designed for multiple disconnected graphs in one instance. For this reason, the structure identifier had to be spread in all the nodes that belong to the same glycan. The duplication of information in the database implies an increased use of memory, which can be a problem for large datasets. This problem can be addressed but any corresponding solution requires extra nodes or properties leading to the same issue.

RDF shows more flexibility in terms of provided API. Different java libraries like Jena or Sesame are available for connecting a java application with every triple store. Moreover, both libraries provide an in memory triple store that can be used for testing purposes. In our study we have used both libraries and there are well documented and easy to use. Virtuoso, like Blazegraph and Jena Fuseki, runs as a standalone server with a useful web interface for managing configuration parameters.

The main obstacle to design substructure search software with RDF has been the ontology definition. Building up ontology for converting glycan structures into triples has been the most time consuming part. It involves reflecting on how to spread the information through different triples in a way that each piece is easy to retrieve and every software requirement is fulfilled. In contrast, defining a specific ontology for substructure search allows storing multiple disconnected graphs in the same database without losing performance.

The shared API and SPARQL, the common query language, made it possible to run four different RDF triple stores without changing our application code but only choosing the right connection driver.

In conclusion, Property Graph databases provide a ready to use solution for substructure search whereas RDF needs a specific ontology for tackling the problem. The development of an ontology can be challenging and time consuming but provides a more flexible solution.

#### Cross Platform Issues

We tested both substructure search implementations under Linux and Windows environments with the same dataset of glycan structures.

Using the Neo4j implementation hardly any difference in terms of query speed and size of results was observed. As Neo4j is implemented in Java, it behaves in the same way on all the systems that can run a Java Virtual Machine, the same situation for Sesame, Jena Fuseki and Blazegraph. However, the Virtuoso implementation produces different query results in different environments. The structures retrieved in the Windows environment were often a subset of the ones retrieved under Linux. In order to establish which answer was correct all results were checked manually notably all the structures retrieved in the Linux environment were correct and in agreement with the Neo4J results. This may mean that Virtuoso triple store possibly contains inconsistencies between the Windows and the Linux version.

We have found a second obstacle during the test with the second query set. In this case, Linux and Windows implementations either crashed after query submission or they caused a crash or a freeze of the machine itself. We could not test the second query set despite several attempts to fix the problem. The novelty of RDF and Property Graph databases technologies can easily explain the presence of issues in the code. Only a large community of users over several years of development will help resolve this type of problems.

## Conclusion

In this paper, we delineated two strategies for substructure searching using new technologies like Property Graph and RDF triple store databases. Using two specific sets of substructures we document that in all the cases RDF has been faster than Property Graph and the gap is increasing with the size of the query. This led us to conclude that our model with Blazegraph RDF Triple Store reduces the query response time that remains close to one second in all the queries of both tested sets. Qualitative measures were discussed to provide the reader with additional criteria for selecting the appropriate technology. Overall, even though both technologies show advantages and disadvantages related to specific qualitative measures, the general lack of standards related to Property Graphs can play a key role in choosing between RDF triple stores and Property Graph databases. In the specific case of substructure search, Property Graphs were seen as a ready-to-use solution for prototyping software. However, when performance and interoperability between different resources is considered, an RDF triple store appears as a more efficient technology.

This study lays the foundation to a possible web application for substructure search. The proposed model is destined to evolve and include a wider set of biological properties in future versions.

## Supporting Information

S1 File Epitope queries. The file contains the SPARQL and Cypher queries for the epitopes of Table 1.
(TXT)

S1 Table Comparison results on the first set of query. Summary of the results obtained using RDF and Property Graph implementations.
(XLSX)

S2 Table Comparison results on the second set of query. Summary of the results obtained using RDF and Property Graph implementations.
(XLSX)

S3 Table Blazegraph results. Query response times obtain using the Blazegraph implementation. The query structure IDs refer to S8 and S7 Tables.
(XLSX)

S4 Table Jena Fuseki results. Query response times obtain using the Jena Fuseki implementation. The query structure IDs refer to S8 and S7 Tables.
(XLSX)

S5 Table Neo4j results. Query response times obtain using the Neo4j implementation. The query structure IDs refer to S8 and S7 Tables.
(XLSX)

S6 Table Sesame results. Query response times obtain using the Sesame implementation. The query structure IDs refer to S8 and S7 Tables.
(XLSX)

S7 Table Openlink Virtuso results. Query response times obtain using the Openlink Virtuoso implementation. The query structure IDs refer to S8 Table and S7 Table.
(XLSX)

S8 Table First set of query structures. List of 128 query relevant biological epitopes used in this study. For completeness, we provide here the GlycoCT encoded structure, the 2d image (cfg format) and the size, in terms of residues, of each epitope. These structures are mainly very short and short according to the partition explained in the paper.
(XLSX)

S9 Table Second set of query structures. List of 60 query epitopes randomly picked up from GlycomeDB. For completeness, we provide here the GlycoCT encoded structure and the size, in terms of residues, of each epitope. These structures are mainly large and very large according to the partition explained in the paper. For limiting the size of the file, 2d images are not provided for this specific dataset.
(XLSX)

S10 Table Dataset composition. Further information about the size of the structures contained in the dataset.
(XLSX)

S11 Table Monosaccharide and substituent. A complete list of all the monosaccharides and substituents used during the study.
(XLSX)
